# Ex vivo γH2AX assay for tumor radiosensitivity in primary prostate cancer patients and correlation with clinical parameters

**DOI:** 10.1186/s13014-022-02131-1

**Published:** 2022-10-05

**Authors:** Ioana M. Marinescu, Manuel Rogg, Simon Spohn, Moritz von Büren, Marius Kamps, Cordula A. Jilg, Elena Fountzila, Kyriaki Papadopoulou, Lara Ceci, Alisa Bettermann, Juri Ruf, Matthias Benndorf, Sonja Adebahr, Daniel Zips, Anca L. Grosu, Christoph Schell, Constantinos Zamboglou

**Affiliations:** 1 Department of Radiation Oncology, Faculty of Medicine, Medical Center – University of Freiburg, University of Freiburg, Freiburg, Germany; 2 Institute for Surgical Pathology, Medical Center – University of Freiburg, Freiburg, Germany; 3 Department of Urology, Faculty of Medicine, Medical Center – University of Freiburg, University of Freiburg, Freiburg, Germany; 4 Second Department of Medical Oncology, Euromedica General Clinic of Thessaloniki, Thessaloniki, Greece; 5 Greece and European University Cyprus, Engomi, Cyprus; 6 Laboratory of Molecular Oncology, Hellenic Foundation for Cancer Research, Aristotle University of Thessaloniki, Thessaloniki, Greece; 7 Department of Nuclear Medicine, Faculty of Medicine, Medical Center – University of Freiburg, University of Freiburg, Freiburg, Germany; 8 Department of Radiology, Faculty of Medicine, Medical Center – University of Freiburg, University of Freiburg, Freiburg, Germany; 9 German Cancer Consortium (DKTK), Partner Site, Freiburg, Germany; 10 Medical Faculty and University Hospital, Radiation Oncology, Eberhard Karls University Tübingen, Tübingen, Germany; 11 German Cancer Consortium (DKTK), Partner Site Tübingen, German Cancer Research Center (DKFZ), Heidelberg, Germany; 12 Berta-Ottenstein-Programme, Faculty of Medicine, University of Freiburg, Freiburg, Germany; 13 Tumorbank Comprehensive Cancer Center Freiburg, Medical Center—University of Freiburg, Faculty of Medicine, University of Freiburg, University of Freiburg, Freiburg, Germany; 14 German Oncology Center, European University Cyprus, Limassol, Cyprus

**Keywords:** Prostate cancer, Intrinsic radio sensitivity, γH2AX foci, Standardized uptake values, Radiotherapy

## Abstract

**Backround:**

Accurate surrogate parameters for radio resistance are warranted for individualized radiotherapy (RT) concepts in prostate cancer (PCa). The purpose of this study was to assess intertumoral heterogeneity in terms of radio resistance using an ex-vivo γH2AX assay after irradiation of prostate biopsy cores and to investigate its correlation with clinical features of respective patients as well as imaging and genomic features of tumor areas.

**Methods:**

Twenty one patients with histologically-proven PCa and pre-therapeutic multiparametric resonance imaging and prostate-specific membrane antigen positron emission tomography were included in the study. Biopsy cores were collected from 26 PCa foci. Residual γH2AX foci were counted 24 h after ex-vivo irradiation (with 0 and 4 Gy) of biopsy specimen and served as a surrogate for radio resistance. Clinical, genomic (next generation sequencing) and imaging features were collected and their association with the radio resistance was studied.

**Results:**

In total 18 PCa lesions from 16 patients were included in the final analysis. The median γH2AX foci value per PCa lesion was 3.12. According to this, the patients were divided into two groups (radio sensitive vs. radio resistant) with significant differences in foci number (*p* < 0.0001). The patients in the radio sensitive group had significantly higher prostate specific antigen serum concentration (*p* = 0.015), tumor areas in the radio sensitive group had higher SUV (standardized uptake values in PSMA PET)-max and -mean values (*p* = 0.0037, *p* = 0.028) and lower ADC (apparent diffusion coefficient-mean values, *p* = 0.049). All later parameters had significant (*p* < 0.05) correlations in Pearson’s test. One patient in the radio sensitive group displayed a previously not reported loss of function frameshift mutation in the *NBN* gene (c.654_658delAAAAC) that introduces a premature termination codon and results in a truncated protein.

**Conclusion:**

In this pilot study, significant differences in intertumoral radio resistance were observed and clinical as well as imaging parameters may be applied for their prediction. After further prospective validation in larger patient cohorts these finding may lead to individual RT dose prescription for PCa patients in the future.

## Backround

Prostate cancer (PCa) still remains the most common type of cancer diagnosed in men in the western world [1] and almost 40% of men aged older than 65 years undergo radiation therapy (RT) as a curative therapy [2]. PCa patients with primary whole gland radiation therapy (RT) have a 10–30% probability of biochemical relapse [3]. New RT concepts, such as focal dose escalation [4] or escalation of systemic therapy [5] improve the outcomes for high-risk non-metastatic PCa patients. RT management decisions are usually based on three pre-therapeutic factors: Prostate Specific Antigen (PSA) serum concentration, Gleason Score (GS) and tumor stage (T stage). They currently represent the most common prognostic factors for the patient’s outcome and are therefore routinely used in the risk stratification of PCa patients [6]. However, none of the latter risk factors was described to correlate with the intrinsic radio sensitivity of PCa lesions [7]. The intrinsic radio sensitivity plays a major role in the therapeutic response to RT and its characterization and quantification might enable individual dose prescription concepts on a lesion or even on a voxel level in the future.

Ionizing radiation (IR) targets mainly the chromosomal DNA and induces DNA breaks directly and indirectly through water radiolysis products. The most common DNA damage patterns due to RT are double-strand breaks (DSBs) [8]. One sensitive tool for measuring the amount of DNA DSBs and therefore quantifying the impact of radiotherapy is detecting the γH2AX foci, a histone which becomes rapidly phosphorylated after exposition to IR [9].

Previous studies [7, 10, 11] established a method to distinguish between radio sensitive and radio resistant PCa lesions, quantifying residual γH2AX foci in ex vivo irradiated tumor samples. Their results indicated a high inter-lesion heterogeneity for intrinsic radio response, suggesting the necessity of personalized RT methods. Following the same methodology, we collected three biopsies from PCa patients during High Dose Rate Brachytherapy (HDR-BT) procedure. In two biopsy cores, the amount of the γH2AX residual foci was determined after ex-vivo irradiation with 0 and 4 Gy for comparison of inter-lesional differences in radio sensitivity. The third core was used for genomic analyses within the PCa tissue. Lastly, the correlation between different clinical parameters, imaging parameters and gene mutations with radio response was examined.

## Material and methods

### Patient population

Twenty one patients with biopsy proven and visible PCa in the pre-therapeutic imaging (magnetic resonance imaging: MRI and prostate-specific membrane antigen prositrin emission tomography: PSMA PET), who had a planned HDR-BT combined with external-beam RT were enrolled. Exclusion criteria were described as the presence of small cell carcinoma/neuroendocrine carcinoma, low-risk PCa according to NCCN criteria and prior irradiation in the pelvis. In total, samples from seven different patients were excluded due to (i) failed labeliling with pimonidazole (two patients) and (ii) lack of tumor or insufficient tumor cells in the samples (five patients). Therefore, sixteen patients with biopsies from 18 PCa foci were included in the current analysis.

All patients received mpMRI (14 patients received a 3T mpMRI and 2 patients a 1.5T mpMRI) and an PSMA-PET/CT (14 patients received a [^18^F] PSMA-1007-PET and 2 patients a [^68^Ga] PSMA-11 PET) prior to therapy. Please see our previous publications for our PET and mpMRI imaging protocols [12, 13]. The study was approved by the Ethics Committee of the Medical Faculty of the University of Freiburg (Nr 274-18_v5). All patients signed informed consent. Further information on the patients and their respective characteristics is given in Table 1. This study represents a preliminary evaluation, illustrating the data of the first 21 enrolled patients, out of a total of 50 anticipated. Under consideration of the labor-intensive workflow of the project, this analysis was performed to assess the feasibility and to obtain preliminary results.

**Table 1:** Patients’ characteristics

Patients, *n*	16
Patients with unilateral lesions, *n*	14
Patients with bilateral lesions, *n*	2
Number of PCa lesions analyzed, *n*	18
Median age in years (range)	70.5 (61–79)
Median PSA before imaging in ng/ml (range)	6.42 (2.1–27.9)
Patients with PSMA PET/CT, *n*	16
18F PSMA PET	14
68 Ga PSMA PET	2
Patients with mpMRI, *n*	16
1.5 T	2
3 T	14
Gleason score in biopsy cores, *n*	
6	2
7a	7
7b	3
8	4
9	0
10	0
Patients with previous ADT, *n*	8

*ADT*, androgen deprivation therapy; *mpMRI*, multiparametric magnetic resonance imaging; *PCa*, prostate cancer; *PSA*, prostateserum antigen; *PSMA PET*, prostate-specific membrane antigen positron emission tomography; *T*, Tesla

### Sample collection

Three different biopsy specimens from each separate tumor lesion were collected during HDR-BT session via trans perineal TRUS/MRI/PET-fusion biopsy, as described by Zamboglou et al. [14]. The specimens were collected before the insertion of the brachytherapy needles. The tumor volume based on PET and MRI was delineated using Eclipse v15.1 software (Varian Medical Systems, USA) before HDR-BT by using validated contouring approaches [16]. Additionally, two distinct image features [15] were acquired per imaging modality: standardized uptake values (SUV-mean/-max) and apparent diffusion coefficient values (ADC-mean/-max). The patients receiving [^68^Ga] PSMA-11 PET were not included in the SUV-analysis. Three patients had no diffusion weighted MRI sequence and were therefore not included in the ADC-analysis. Two of them consequently had no contouring of the GTV-MRI.

The three biopsy samples were retrieved as fine needle biopsies with a reusable biopsy gun (Uromed REF6020) with trocar-shaped biopsy needles (Uromed REF 6025.10) [16] for: (i) 0 Gy irradiation, (ii) 4 Gy irradiation and (iii) next generation sequencing (NGS). Two patients presented bilateral tumor lesions and in these 6 probes were therefore collected from the 2 different tumor areas, respectively.

After collection, the NGS sample was stored directly into 4% formaldehyde (24 h) and subsequently into 70% ethanol before being embedded into paraffin. The two samples meant for IR were placed in Petri dishes containing 10 mL DMEM medium supplemented with 10% fetal calf serum, 2% HEPES buffer, 1% antibiotics, 1% sodium pyruvate and 1% non-essential amino acids (all Biochrom AG, Berlin, Germany) and cultivated (37 °C, 95% humidity and 5% CO_2_) for 22 h [12]. Subsequently, the hypoxic marker pimonidazole (Hypoxyprobe Inc, hpi, Middlesex, Burlington, USA) was added to the samples for 2 h (37 °C, 95% humidity and 5% CO_2_). After the ex vivo irradiation (with 4 and respectively 0 Gy) the medium was exchanged, the samples were further cultivated (24 h) and afterwards fixated in 4% formaldehyde (24 h). Finally, the samples were stored in 70% ethanol before further processing for paraffin embedding (FFPE samples) applying standard diagnostic procedures at the department for pathology.

#### Staining and imaging of tumor specimens

For each sample four different 2 μm thick cross-sections were cut from the paraffin-embedded tumor material using a microtome and transferred to slide.

Firstly, all slides were incubated overnight at 42 °C. Afterwards, the sections were deparaffinized (xylol, 30 min), rehydrated (graded alcohol series), washed in PBS (1 min), steam cooked in in pH6 citrate buffer for epitope retrieval, cooled on ice (15 min) and washed in PBS for another 5 min as described by Menegakis et al. [12]. Afterwards, the samples were stained for:

- Residual foci analysis with anti-γH2AX (anti-phospho-Histone H2A.X, Ser139, 05-636, Merck Millipore, Clone JBW301, dilution 1:100) and anti-AMACR (Alpha-Methylacyl-CoA Racemase) for tumor identification (Anti-Human AMACR Clone 13H4, IR06061-2, Dako Omnis/Agilent) in AMACR ready-to-use antibody solution and counterstained with Hoechst 33,342 (H3570, Thermo Fisher Scientific, Inc. Dilution 1:1000).
- Identification of the proliferative areas with anti-KI-67 ready-to-use solution (KI-67/MIB1, IR62661-2, Dako Omnis/Agilent).
- Recognition of the hypoxic areas with anti-pimonidazole (mouse monoclonal, Natural Pharmacia International, Belmont, MA, USA, dilution 1:100) with ARKTM Kit (animal research kit; Dako Deutschland GmbH, Hamburg, Germany) and VECTAstain Kit, respectively (Vectastain Elite ABC kit, PK-6102, Mouse IgG, Vector Laboratories, Inc., 30 Ingold Road, Burlingame, CA 94,010 USA)For the γH2AX foci visualization, the sections were first blocked in 5% BSA in PBS (1 h), followed by incubation with primary antibodies (2 h), washing with PBS and afterwards, incubation with secondary fluorophore-tagged antibodies (anti-mouse IgG, Alexa Fluor 555, A31570, Thermo Fisher Scientific Inc., dilution 1:400) and secondary AMCR antibodies (anti-rabbit IgG, Alexa Fluor 647, A-21245, Thermo Fisher Scientific, Inc., dilution 1:500) for 45 min. Finally, after repetitive washing in PBS, the sections were mounted in ProLong Gold Antifade (P36930, Thermo Fisher Scientific, Inc.).

For both the KI-67 staining samples were blocked in 5% BSA in PBS (1 h) and peroxidase-blocking solution (S202386-2, DAKO Omnis/Agilent). Primary antibodies were diluted in BSA blocking solution and incubated for 2 h. The EnVision FLEX + Mouse (LINKER) Kit (K802121-2, Dako Omnis/Agilent) or anti-goat HRP (P0449, Dako Omnis/Agilent, 1:500) secondary antibodies were applied, respectively. The DAB + Substrate Chromogen System (Dako Omnis/Agilent) was used for immunohistochemistry (IHC). Finally, the slides were counterstained with Hematoxylin (105,174, Merck) and mounted in Entellan. The same protocol was used for the Pimonidazole staining as described by Menegakis et al. [12].

For immunofluorescence evaluation a Zeiss Axio Observer 7 microscope (Colibri 7 illumination system, Axiocam 702 mono camera, ApoTome.2 device, motorized scanning stage; 40 × objective and 49 DAPI, 43 HE dsRed and 50 Cy5 filter sets) controlled by ZEN 3.1 (blue edition) software (Carl Zeiss, Jena, Germany) was used. IHC stained sections were scanned with a digital color camera at 40-fold magnification (Ventana DP 200 slide scanner, Roche Diagnostics Deutschland GmbH, Mannheim, Germany) and positive areas were marked in the scan. KI-67/Pimonidazole negative tumor areas were selected from the respective immunofluorescence sections for analysis. Z-stack images (0.25 µm steps) were taken for better visualization and quantification of γH2AX foci within cell nuclei.

After identification of tumor areas (AMACR positive areas) 50 tumor cells were randomly selected from each sample (sham-irradiated and 4 Gy irradiated). Cells showing pan-nuclear staining, mitotic, necrotic or apoptotic cells were excluded from the analysis. Finally, the area of each nucleus was measured and the number of foci in each nucleus was manually counted.

#### Next generation sequencing

Targeted NGS was employed to investigate the mutational profile of patients with available FFPE tissue material. FFPE tissue evaluation and processing, DNA extraction and NGS were implemented in the Laboratory of Molecular Oncology (MOL Hellenic Foundation for Cancer Research/HeCOG/AUTH), located in Thessaloniki, Greece.

Hematoxylin & eosin-stained sections from the patients’ tissue blocks were assessed by a pathologist for tumor presence and marking of tumor dense areas. Tumor DNA was extracted from the marked areas upon manual macrodissection of 10 μm unstained FFPE sections, using the QIAamp DNA Mini Kit (Qiagen GmbH, Hilden, Germany). Tumor cell content (TCC) was assessed as an approximate metric for tumor DNA in the extracted samples, corresponding to tumor nuclei vs. all nuclei in the areas marked for macro-dissection. The FFPE tissue blocks from nine patients had adequate tumor cell content and were processed for DNA extraction and subsequent tumor genotyping with NGS.

For NGS analysis, a custom Ampliseq panel (IAD207308_231; Ion Torrent/ThermoFisher Scientific, Paisley, UK) was applied targeting coding relevant regions of 64 genes, including various DNA damage response and repair genes and others that are members of signaling pathways and/or with potential clinical impact as drug targets in prostate cancer, including Wnt pathway, PI3K, cell cycle and RAS/MAPK components [17–20]. Panel design was based on the human GRCh37 assembly and covered ~ 119.21 kb with 1149 amplicons.

For library construction, a multiplex PCR was performed using 20 ng DNA per sample and the Ampliseq primers along with the Ampliseq Library Kit v.2.0 and Ion Xpress barcodes, according to the manufacturer’s instructions (Life Technologies, Carlsbad, CA). Resulting libraries, were clonally amplified on the One-Touch-2 instrument, enriched on the OneTouch ES with the Ion PI template OT2-200 Kit v.3 and sequenced on the Ion Proton with the Ion PI Sequencing 200 Kit v.3 (Life Technologies, Carlsbad, CA).

Data retrieval, base and variant calling were performed on the Torrent Server using Torrent Suite v5.10.0, followed by variant annotation with the Ion Reporter software (version 5.18) to automatically annotate single nucleotide variants (SNVs), multiple nucleotide variants (MNVs) and small insertions/deletions (INDELs). Variants were evaluated for amplicon reads and quality filtered to accept eligible variants with more than 100 amplicon reads; *p*-value < 0.0001; position coverage > 100; variant coverage > 40 (when position coverage 100–200); ± strand bias > 10% for position and variant coverage. Non-annotated variants and indels with G-stretches were excluded from analysis. Only variant allele frequencies (VAF) of > 5% were accepted. Based on standard quality metrics, the libraries for the nine patients’ samples were technically efficient, yielding high quality mapped reads, aligned to panel target regions. Specifically, median mean depth of sequenced tumor samples was 4256 (mean: 4243; min–max: 3094–5418) and median uniformity was 96.7% (mean: 96.1%; min–max: 92–97.4%), whereas 589 out of 599 retrieved variants (98.3%) were considered eligible for the aforementioned samples and called mutations if amino acid or splice site changing with no reported minor allele frequency (MAF) or with MAF < 0.1% according to 5000 Exomes database if annotated SNPs. Mutations were considered pathogenic according to ClinVar database.

### Statistical analysis

The statistical analysis was performed with GraphPad Prism v8.4.2 (GraphPad Software, USA). Normal distribution was tested using the D’Agostino-Pearson omnibus normality test. For inter- -patient analysis a paired Mann Whitney test was used for comparison (two-tailed). Correlation between γH2AX foci number and clinical variables was assessed with Peaesons’s correlation test. The significance level for all tests was defined as 0.05.

The normalized γH2AX foci number (nfoci) was adjusted as described by Menegakis et al. [13] using the following formula:

$$nfoci = (Area_{m} /Area_{i} )\times Nfoci - cfoci_{{OGy }}$$

where ${\text{Area}}_{{\text{i}}}$ and ${\text{Area}}_{{\text{m}}}$ represent the measured area of each nucleus selected for residual γH2AX analysis and the mean nuclei area from each patient, respectively. Nfoci is the actual number of residual foci counted in each nucleus and ${\text{cfoci}}_{{{\text{OG}}_{{\text{y}}} }}$ amounts to the mean number of residual γH2AX foci in the sham-irradiated group of each patient. The value of the normalized γH2AX foci number was set as zero, if the subtraction was leading to a negative number. [11] For one patient, due to insufficient tumor cells in the sham irradiated sample, the γH2AX foci value was calculated without subtracting the mean number of residual γH2AX foci in the sham-irradiated sample (${\text{cfoci}}_{{{\text{OG}}_{{\text{y}}} }}$).

## Results

**Fig. 1:**
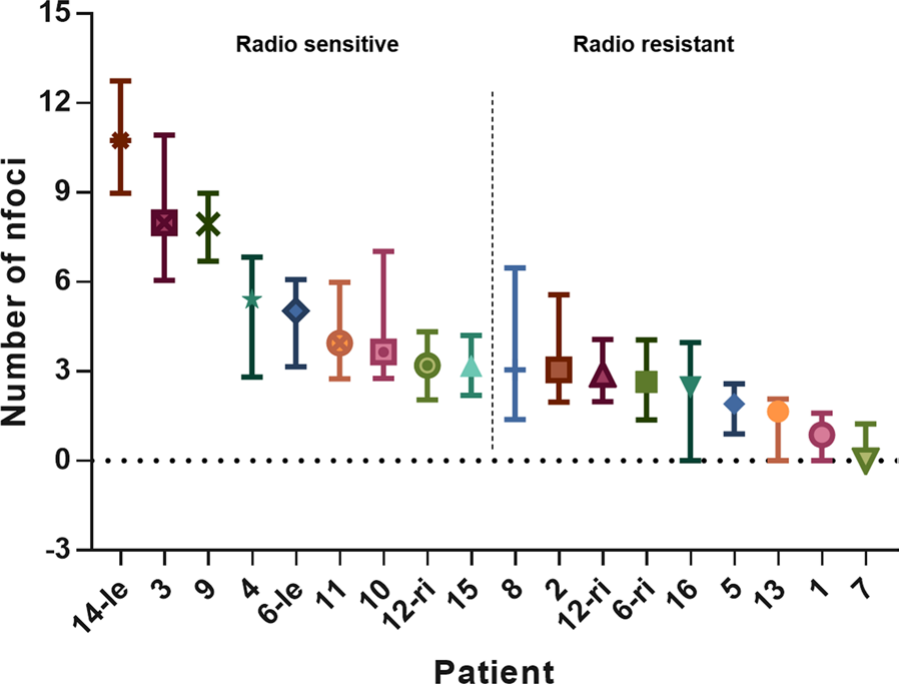
Distribution of the residual үH2AX nfoci values in the different patients arranged in descending order from left to right. The median nfoci value of each individual patient and 95% confidence intervals of the median value estimation are shown. Two subgroups were created according to the median nfoci value between all patients (3.12). The dotted line shows the median value of residual үH2AX foci in all patients. Abbreviations: ri: right, le: left

**Fig. 2:**
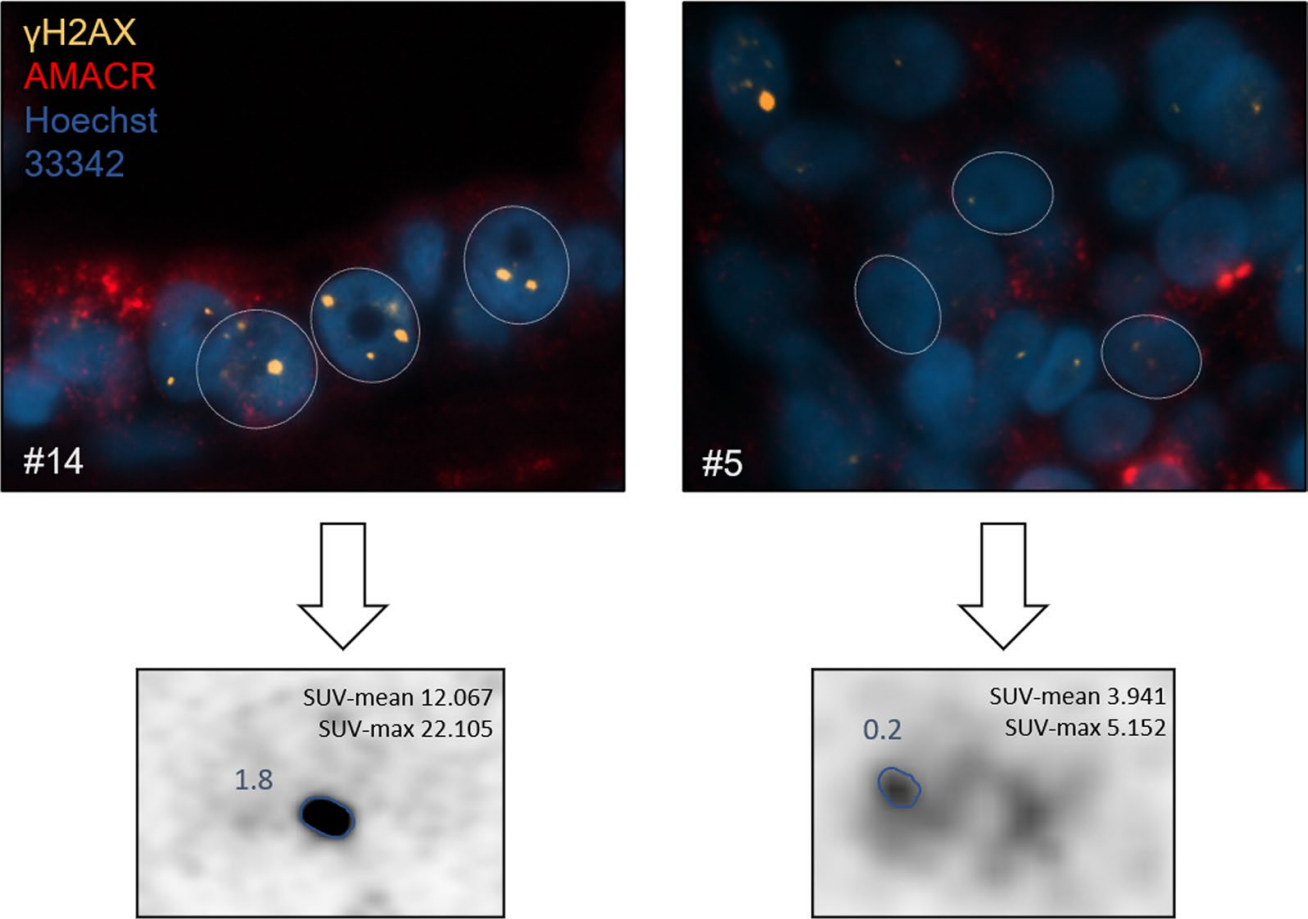
Immunofluorescence image of γH2AX foci distribution in PCa patients and corresponding PSMA PET images. Immunofluorescence images from two patients are shown. Patient #14 was classified as radio sensitive, whereas patient #5 as radio resistant. DNA DSB marker γH2AX foci is presented in yellow (Alexa 555), DNA counterstain (in blue) was used to visualize the cell nuclei (Hoechst 33,342) and AMACR in red for PCa visualization. For each patient the PSMA PET scans are shown. The GTVs are marked with blue, respectively. SUV-mean/max values are shown. Abbreviations: PCa: prostate cancer; DNA DSB: double strand breaks; AMACR: AlphaMethylacylCoA Racemase; GTV: gross tumour volume; PSMA PET: positron emission tomography with rostate-specific membrane antigen

**Fig. 3:**
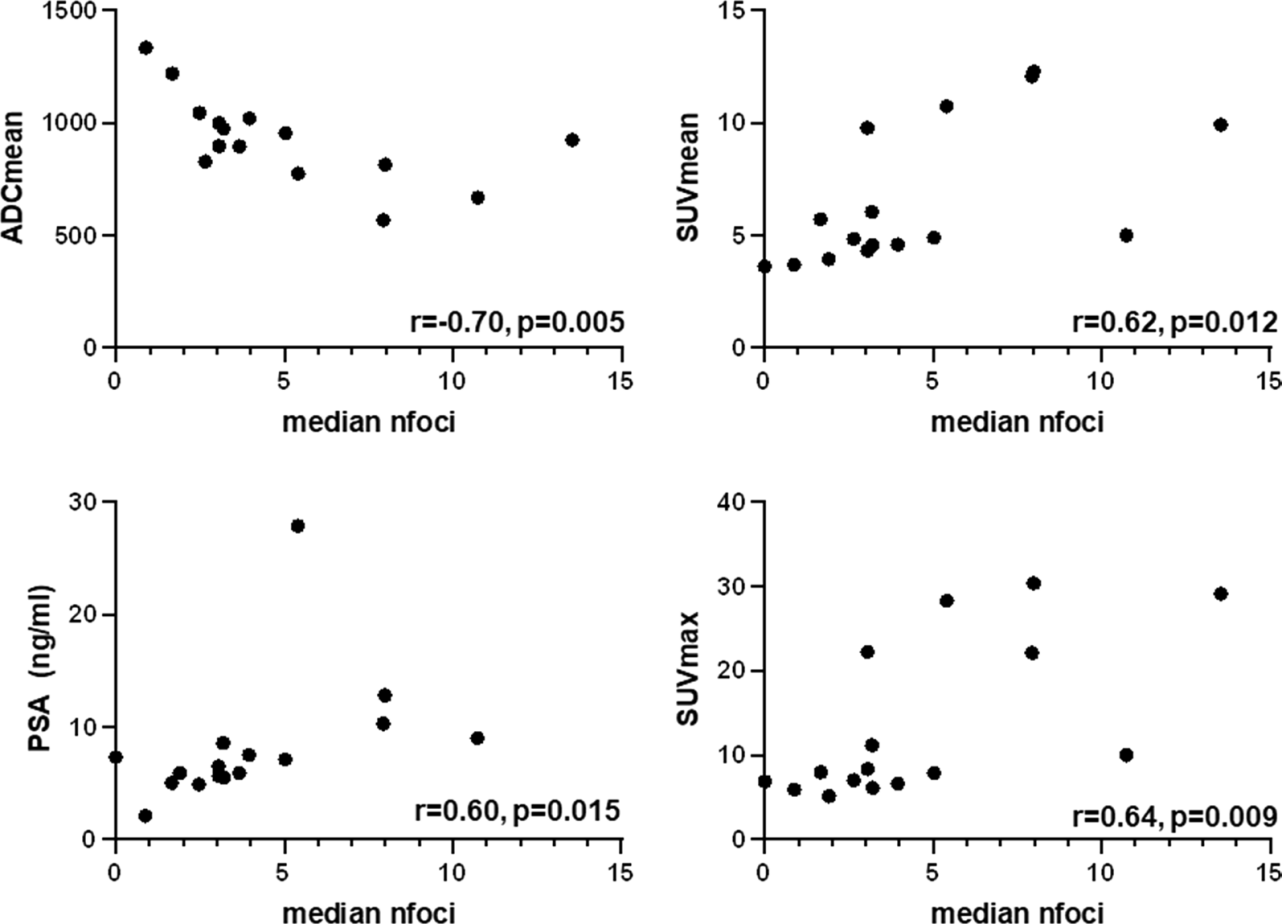
Pearson’s correlation test between the median yH2AX foci number per tumor with clinical and imaging parameters. The results of the Pearson’s correlation test between median yH2AX foci number per tumor with clinical and imaging parameters are represented. Abbreviations: nfoci: median yH2AX foci number per PCa lesion, SUV: standardized uptake value, ADC: apparent diffusion coefficient

Figure 1 shows the distribution of residual γH2AX foci for the 18 PCa lesions, 24 h after ex-vivo irradiation. The median value of the normalized γH2AX foci number for all patients was calculated (3.12, range: 0–10.74) and the patients were divided into 2 groups according to this value: radio resistant vs radio sensitive. The median normalized γH2AX foci value was 5.03 (range: 3.18–10.74) and 2.5 (range: 0–3.06) for the radio sensitive and radio resistant group, respectively (*p* < 0.001). Two patients showed two different bilateral tumor areas, respectively. In one patient, one lesion was perceived as radio sensitive (median normalized γH2AX foci: 5.03) whereas the contralateral biopsy core was relocated to the radio resistant group (median value: 2.64). The other patient showed no difference in intrinsic radio sensitivity between the two tumor areas (*p* = 0.572). Figure 2 shows the heterogeneous response to RT between the patients and lesions. To investigate the possible predictive markers for radio sensitivity and radio resistance, we proofed the association of the acquired clinical and genetic imaging factors to each group (Table 2). No significant difference between the two groups was observed regarding age, GS, previous ADT, cT stage, absolute GTV volume and ADC-max values. The PSA serum concentration as well as the SUV-max and SUV-mean values were significantly higher in the radio sensitive group. ADC-mean values were significantly smaller in the radio sensitive group. Figure 3 shows the results of the Pearson’s correlation test for the significant parameters.

**Table 2:** Distribution of the clinical and radiomic variables

Factor	Median value/*n*		*p* value
	Radio-sensitive group	Radio-resistant group	
Age (years)	70	71	0.814
PSA serum concentration (ng/ml)	7.5	5.63	0.015
GTV-PET (ml)	1.95	1.6	0.593
GTV-MRI (ml)	0.8	0.8	0.816
SUV-max	16.65	6.97	0.037
SUV-mean	7.99	4.44	0.028
ADC-min	1589	1766	0.437
ADC-mean	895	1022	0.049
ADT (n)	4	4	> 0.999
*GS*			> 0.999
6	1	1	
7a	4	4	
7b	2	2	
8	2	2	
9	0	0	

cT stage	PSMA PET/MRI	PSMA PET/MRI	
T2a	0/1	2/1	0.176 (PSMA PET)
T2b	0/0	0/0	
T2c	2/0	2/1	
T3a	1/5	2/3	0.705 (MRI)
T3b	4/1	0/0	
T4	0/0	0/0	

*ADC*, apparent diffusion coefficient; *ADT*, androgen deprivation therapy; *GTV*, gross tumor volume; *MRI*, magnetic resonance imaging; *PET*, positron emission tomography; *PSA*, prostateserum antigen; *SUV*, standardized uptake values

**Fig. 4:**
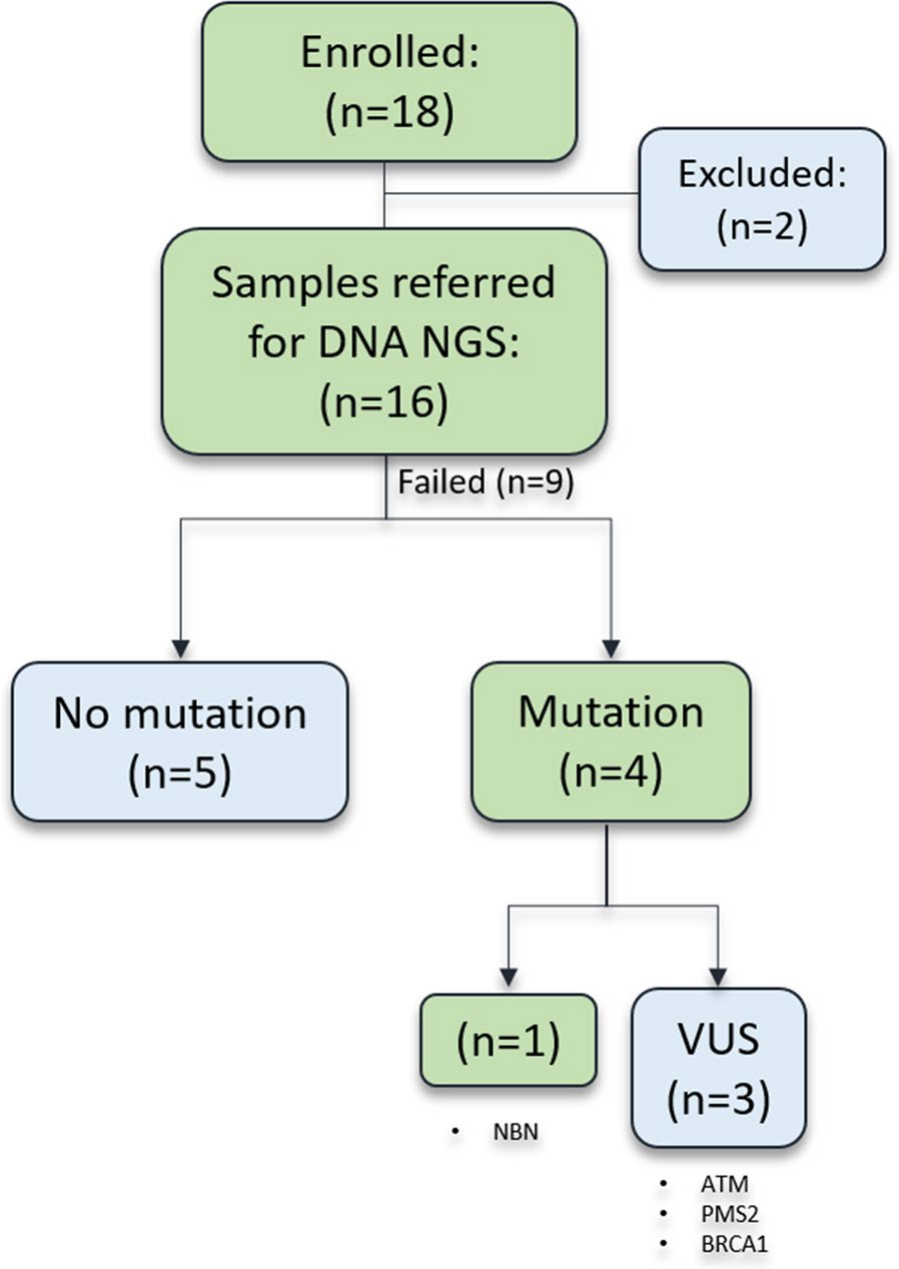
Next generation sequencing of PCa samples. Abbreviations: DNA NSG: DNA Next Generation Sequencing; VUS: variant of unknown significance

The tumor DNA samples from nine patients were analyzed with NGS. In turn, the tumors of four patients possessed mutations. However, three patients carried mutations in *ATM*, *BRCA1* and *PMS2* genes that were classified as a variant of unknown significance (VUS), according to the ClinVar database. Finally, only one patient displayed a previously not reported loss of function frameshift mutation in the *NBN* gene (c.654_658delAAAAC) that introduces a premature termination codon and results in a truncated protein (p.Lys219AsnfsX16). The *NBN* c.657_661delACAAA mutation that results in the same truncated protein as the one reported herein has been reported as pathogenic in the ClinVar database, is a founder variant among Central and Eastern European populations and has been associated with breast and PCa [21, 22] (Fig. 4).

For the cT classification three patients were excluded (6 and 12, with bilateral lesions and where ADC sequence was not available). cT staging was conducted via PSMA PET and mpMRI, respectively.

## Discussion

In the current study, we used the γH2AX assay, established by the group of Menegakis et al. [12] to determine the intra- and inter-tumoral heterogeneity regarding intrinsic radio sensitivity in PCa patients.

Current RT methods are characterized by a homogeneous dose distribution within the prostate. However, biochemical recurrence free survival (bRFS) has been shown to be improved by focal dose escalation based up to 7% for intermediate- and high-risk prostate cancer without impacting toxicity and quality of life [5]. An individualized dose prescription according to the respective radio resistance of each tumor lesion could be a further step towards individualized RT in PCa. In our study we found significant differences in radio resistance between PCa lesions in different patients. Interestingly, also a difference in radio resistance was found within two PCa lesions of the same patient. Our results are similar with the observations by DeColle et al. [14]. Taken together the current evidence suggests the necessity of individual RT concepts, due to the inter-tumoral heterogeneity in intrinsic cellular radiation sensitivity. However, quantification of radio resistance by measurement of residual γH2AX foci may not be suitable for clinical routine due to its labor-intense nature. Likewise, clinical surrogate parameters are warranted for its prediction.

Therefore, we investigated the possible predictive characteristics of the patients’ clinical and imaging variables for radio resistance. No significant difference was observed between intrinsic radio sensitivity of PCa samples and cT stage, PCa lesion volume, ADT admission or GS, which is consistent with the results of De Colle et al. [14]. However, our results showed significantly higher PSA serum concentrations in the radio sensitive group than in the radio resistant patients and strong negative correlation in Pearson’s test. Apart from its diagnostic and prognostic features, PSA has been showed to have an important role in PCa proliferation [23] and it is well known that radiation sensitivity increases with proliferation due to telomere dysfunction [24]. Additionally, SUV-max and SUV-median values in PSMA PET images were significantly higher in the radio sensitive group, whereas ADC-median values were significantly smaller. All values showed also a significant correlation with nfoci number with r ≥ 0.60 in Pearson’s test. This suggests a higher PSMA expression and a higher cell density in radio sensitive PCa lesions. Nevertheless, first studies reported that high SUV-max values in primary PCa lesions are associated with an increased relapse rate after surgery [25]. These findings must be discussed under consideration of the biological properties of the PSMA protein [26, 27]. PSMA on PCa cells hydrolyzes poly-y-glutamated folates and increases the glutamate and folate concentrations within PCa cells [26]. Additionally, the expression of PSMA correlates with the PI3K-Akt pathway [28]. Both mechanisms are associated with an increase in tumor proliferation. Furthermore, PSMA contributes in tumor angiogenesis [29]. Likewise, it can be assumed that PCa lesions with high PSMA expression might possess a higher metastatic potential and thus a faster systemic disease progression which might explain worse outcomes after RT despite an increased radio sensitivity. After further validation, this observation may change treatment concepts in PCa patients. In patients with high SUV values in PSMA PET imaging, low ADC values in MRI and high PSA levels an escalation of systemic treatment and a reduction of RT dose might increase the therapeutic ratio.

The correlation between mutations in known PCa susceptibility genes (such as *BRCA1* and 2, *ATM*, *PMS2* and *NBN*) and an increased risk for PCa was showed in multiple studies [30–32]. Moreover, mutations in the *NBN*, *BRCA2* and *ATM* genes are associated with a more aggressive PCa phenotype and worse clinical outcome [21, 33, 34]. In the current study, one sample showed a significant *NBN* gene mutation. The PCa specimen was derived from a patient classified as radio sensitive, with a median number of foci of 3.95 and a high risk PCa, according to the clinical parameters. The encoded protein (nibrin) is a component of the *MRE11/RAD50/NBS1 (MRN)* complex, which is thought to be involved in DNA DSB repair and DNA damage-induced checkpoint activation [35]. In a prospective study, Berlin et al. investigated the role of *NBN* gene mutations on the clinical outcome of PCa patients treated with image-guided radiotherapy and radical prostatectomy [36]. The 5 years biochemical relapse free rate in irradiated patients was significantly higher in patients with *NBN* gain mutations compared to neutral gain mutations. However, *NBN* gain did not have any significant prognostic value in the surgery cohort. Therefore, one can speculate that, while *NBN* gain influences the intrinsic tumor radio resistance, *NBN* loss of function mutations may contribute to the sensitization of PCa to radiation. However, more studies need to be carried out in order to investigate the significance of the *NBN* gene mutation on PCa.

In the following, we want to discuss the limitations of our study. First, this pilot study considered only 18 PCa lesions. This is justified by considering the labor-intensive processing required for each patient. An enlargement of the cohort up to 50 patients is currently ongoing. Second, only one biopsy core per PCa lesion was irradiated with 4 Gy and thus intra-tumoral heterogeneity was not accounted for. However, De-Colle et al. demonstrated that one biopsy is sufficient to estimate the mean value of residual γH2AX foci per dose level and account for intra-tumoral heterogeneity [7].

Finally, the time difference between the estimation of the PSA serum concentration and the biopsy core recovery should be considered (median time difference of 69.5 days with a range of 6–205 days). Moreover, SUV-mean and SUV-max and ADC-mean values showed potential predictive features in detecting the eligibility of patients for RT. Nonetheless, the pre-therapeutic images and the acquiring of the biopsies during the first HDR-BT session were not performed with an equal time difference for all patients. The median number of days between the imaging and the first HDR-BT session was 105.5 days (range: 6–180) and 43 days (range: 4–200) for PSMA-PET and mpMRI, respectively.

## Conclusion

In this pilot study we investigated the inter-tumoral heterogeneity in radio resistance in PCa patients and novel radiomic and genomic biomarkers correlated with the intrinsic radio resistance on a tumor level. After further prospective validation in larger patient cohorts, these findings might be used in the future for personalized RT concepts with individual RT dose prescriptions.

## Data Availability

The datasets used and/or analysed during the current study are available from the corresponding author on reasonable request.

## Abbreviations

ADC: Apparent diffusion coefficient
ADT: Androgen deprivation therapy
AMACR: Alpha-methylacyl-CoA racemase
bRFS: Biochemical recurrence free survival
BSA: Bovine serum albumin
CTV: Clinical target volume
DAB: 3,3′-Diaminobenzidine
DNA: Deoxyribonucleic acid
DSB: Double-strand break
FFPE: Formalin-fixed, paraffin-embedded
GS: Gleason score
GTV: Gross tumor volume
HDR-BT: High dose-rate brachytherapy
IHC: Immunohistochemistry
INDELs: Small insertions/deletions
IR: Ionizing radiation
MAF: Minor allele frequency
MNVs: Multiple nucleotide variants
mpMRI: Multiparametric magnetic resonance imaging
NBN: Nibrin
NCCN: National comprehensive cancer network
NGS: Next generation sequencing
PBS: Phosphate-buffered saline
PCa: Prostate cancer
PCR: Polymerase chain reaction
PET: Positron emission tomography
PSA: Prostate specific antigen
PSMA: Prostate-specific membrane antigen
RT: Radiotherapy
SNVs: Single nucleotide variants
SUV: Standardized uptake values
T: Tesla
TCC: Tumor cell content
TRUS: Transrectal utrasound
VAF: Variant allele frequencies
vs: Versus
VUS: Variant of unknown significance
